# Establishing Ultrasound Thresholds for Sarcopenia Diagnosis in Older Brazilian Adults

**DOI:** 10.3390/muscles4040057

**Published:** 2025-11-20

**Authors:** Sérgio Zabotto Dantas, Danielli Candido Munhoz Evangelista, Bruna Zampieri Nogueira Cozza, Marcelo Dib Bechara, Sandra Maria Barbalho, Eduardo Federighi Baisi Chagas, Adriano Cressoni Araújo, Elen Landgraf Guiguer, Camila Maria de Arruda, Juliana da Silva Soares de Souza, Karina Quesada, Cláudia Rucco Penteado Detregiachi

**Affiliations:** 1 Postgraduate Program in Structural and Functional Interactions in Rehabilitation, School of Medicine, Universidade de Marília (UNIMAR), Marília 17.525–902, São Paulo, Brazil; szdmed@hotmail.com (S.Z.D.); dib.marcelo1@gmail.com (M.D.B.); smbarbalho@gmail.com (S.M.B.); efbchagas@unimar.br (E.F.B.C.); adrianocressoniaraujo@yahoo.com.br (A.C.A.); elguiguer@gmail.com (E.L.G.); karinarquesada@gmail.com (K.Q.); 2 Unimagem–Diagnostic Imaging Unit, Beneficent Hospital of the Universidade de Marília (UNIMAR), Marília 17.525–902, São Paulo, Brazil; danielli.candido@unimagemdm.com.br (D.C.M.E.); brunazampnogueira@gmail.com (B.Z.N.C.); 3 Department of Biochemistry and Pharmacology, School of Medicine, Universidade de Marília (UNIMAR), Marília 17.525–902, São Paulo, Brazil; 4 Department of Biochemistry and Nutrition, School of Food and Technology of Marília (FATEC), Marília 17.500-000, São Paulo, Brazil; 5 School of Nutrition, Universidade de Marília (UNIMAR), Marília 17.525–902, São Paulo, Brazil; camilamarianutricao@gmail.com; 6 FAI–Centro Universitário de Adamantina, Adamantina 17.800-057, São Paulo, Brazil; 7 School of Veterinary Medicine, Universidade de Marília (UNIMAR), Marília 17.525–902, São Paulo, Brazil; juliana.silva.soares.souza@gmail.com

**Keywords:** muscle quality, sarcopenia, computed tomography, ultrasound

## Abstract

**Background/Objectives**: Despite the increasing use of ultrasound (US) as a tool for assessing muscle mass and diagnosing sarcopenia, its application remains limited because few studies have validated cut-off points for specific populations. This study aimed to propose US cut-off points for diagnosing sarcopenia in Brazilian individuals aged 60 years and older. **Methods**: Patients schedule for elective abdominal computed tomography (CT) were also evaluated with musculoskeletal US of the thigh. CT images were obtained at the level of the third lumbar vertebra. US measurements included the thickness of the rectus femoris (RF) muscle and the rectus femoris combined with the vastus intermedius (RF + VI). Receiver Operating Characteristic (ROC) curves determine the sensitivity and specificity of the US cut-off points. The area under the curve (AUC) and 95% confidence intervals (CI) were calculated. **Results**: The study sample (n = 88) had a mean age of 71.8 ± 8.7 years, and 64% were women. The proposed cut-off points for diagnosing sarcopenia using US, based on the mean ± SD, were ≤19.1 mm and ≤15.9 mm for RF thickness and ≤31.9 mm and ≤29.2 mm for RF + VI thickness in men and women, respectively. These cut-off points demonstrated good accuracy and significant AUC values. **Conclusions**: This study proposes US-based cut-off points with good accuracy for suggesting sarcopenia diagnosis, particularly when assessing RF thickness.

## 1. Introduction

Globally, populations are aging rapidly due to increased life expectancy and improved healthcare. Consequently, maintaining functional capacity in older adults has become increasingly important [1,2,3].

Skeletal muscle is the largest organ in the human body by mass and a significant contributor to overall health and longevity. Loss of skeletal muscle negatively impacts several physiological parameters, including mobility, respiration, vision, thermoregulation, and metabolic homeostasis [4,5,6]. Skeletal muscle tissue is dynamic, depending on the balance between protein synthesis and degradation, both of which are influenced by various factors, including nutritional status, hormonal balance, physical activity, injury, and disease, among others [7].

Loss of skeletal muscle mass and strength, termed sarcopenia, is a global public health concern [8,9]. This progressive and generalized muscle disorder is associated with an increased risk of a wide range of adverse outcomes, including impaired mobility and high morbidity [8,10], longer hospitalization [11], major postoperative complications [12], poor clinical outcomes after treatments such as cancer therapy [13], and high mortality [14,15].

To date, there is no universal consensus on the definition of sarcopenia. The lack of a single definition has impacted research and clinical practice, generating uncertainty regarding which definitions, cut-off points, and muscle assessment tools to use [9,16,17]. A significant milestone was achieved in 2019 when the European Working Group on Sarcopenia in Older People 2 (EWGSOP2) revised its consensus, updating the definition and diagnostic algorithm for sarcopenia [18]. This updated algorithm incorporates a stepwise approach to “find, assess, confirm, and determine the severity of cases,” aiming to facilitate its use in clinical settings. In this context, imaging assessment is crucial, as it enables early diagnosis of sarcopenia, potentially allowing timely interventions that can delay adverse clinical outcomes, improve quality of life, and reduce public healthcare costs [19,20].

Clinical practice and research employ various qualitative and quantitative methods to determine muscle mass. Different imaging techniques, including dual-energy X-ray absorptiometry (DEXA), computed tomography (CT), magnetic resonance imaging (MRI), and ultrasound (US), are utilized to assess muscle mass and quality, thereby facilitating the diagnosis of sarcopenia [21,22]. However, each method has advantages and limitations, with varying levels of reproducibility and feasibility [22,23,24,25].

CT has been increasingly used in research and as a routine diagnostic tool for assessing muscle quantity and quality [22,26]. It is considered the gold standard for body composition analysis, especially in nutritionally vulnerable patients [27,28]. In most studies, segmentation is performed at the level of the third (L3) or fourth (L4) lumbar vertebra, and regions of interest include the psoas or all muscles present in the slice (paraspinal, psoas, abdominal wall) [22,25,29]. However, CT has limitations that restrict its use, such as cost, time, and radiation exposure.

US is a tool that can assess both muscle quantity and quality. Recently, the European Geriatric Medicine Society proposed a protocol for using US in muscle mass evaluation [30]. Despite the growing use of the US for sarcopenia diagnosis [20,31,32], its application remains limited, mainly due to the lack of studies providing validated cut-off points for specific populations [33], including the Brazilian population (Fu et al., 2023).

In this scenario, the study aimed to propose US cut-off points for diagnosing sarcopenia in Brazilian individuals aged 60 years and above.

## 2. Results

The study sample (n = 88) had a mean age of 71.8 ± 8.7 years, ranging from 60 to 99 years, with 64% female participants. Based on CT, the skeletal muscle area (SMA) and skeletal muscle index (SMI) calculations indicated a prevalence of sarcopenia of 48% (n = 40) and 40% (n = 35), respectively. RF muscle thickness, measured by US, was significantly greater among participants without sarcopenia, according to both SMA and SMI-based assessments (Table 1).

**Figure 1:**
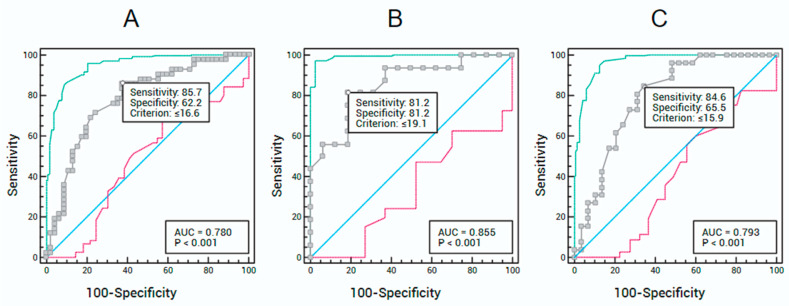
Receiver Operating Characteristic (ROC) curves to determine the sensitivity and specificity of rectus femoris muscle thickness cut-off points measured by ultrasound for sarcopenia diagnosis, compared with skeletal muscle area assessed by computed tomography, for the total sample (**A**), male participants (**B**), and female participants (**C**). The blue line represents the line of nullity, indicating a test with no discriminatory ability (AUC = 0.5). The gray line represents the main ROC curve, which provides the AUC, sensitivity, and specificity for various cutoff points. The green line shows the upper 95% confidence interval, and the red line shows the lower 95% confidence interval.

According to SMA (cm^2^) assessed by CT, for the total sample (Figure 1A), the ROC curve showed an AUC of 78.0% (95% CI: 67.9–86.2%), adopting a US-measured RF thickness cut-off of ≤16.6 mm for sarcopenia diagnosis. This cut-off yielded a sensitivity of 85.7% and specificity of 62.2%. For male participants (Figure 1B), the ROC curve showed an AUC of 85.5% (95% CI: 68.6–95.4%), with an RF thickness cut-off of ≤19.1 mm, resulting in 81.2% sensitivity and 81.2% specificity. For female participants (Figure 1C), the ROC curve showed an AUC of 79.3% (95% CI: 66.3–89.0%), adopting an RF thickness cut-off of ≤15.9 mm, with 84.6% sensitivity and 65.5% specificity.

**Figure 2:**
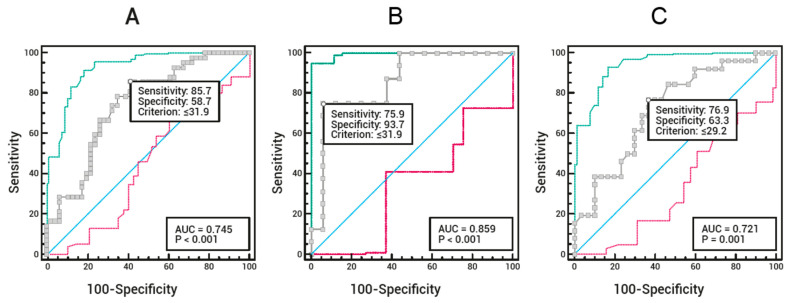
Receiver Operating Characteristic (ROC) curves to determine the sensitivity and specificity of rectus femoris plus vastus intermedius (RF + VI) muscle thickness cut-off points measured by ultrasound for sarcopenia diagnosis, compared with skeletal muscle area assessed by computed tomography, for the total sample (**A**), male participants (**B**), and female participants (**C**). The blue line represents the line of nullity, indicating a test with no discriminatory ability (AUC = 0.5). The gray line represents the main ROC curve, which provides the AUC, sensitivity, and specificity for various cutoff points. The green line represents the upper 95% confidence interval, and the red line represents the lower 95% confidence interval.

According to SMA (cm^2^) assessed by CT, the ROC curve for the total sample (Figure 2A) showed an AUC of 74.5% (95% CI: 64.0–83.2%), adopting a US-measured RF + VI thickness cut-off of ≤31.9 mm for sarcopenia diagnosis. This cut-off yielded a sensitivity of 85.7% and specificity of 58.7%. For male participants (Figure 2B), the ROC curve showed an AUC of 85.9% (95% CI: 69.1–95.6%), with an RF + VI thickness cut-off of ≤31.9 mm, resulting in 75.0% sensitivity and 93.7% specificity. For female participants (Figure 2C), the ROC curve showed an AUC of 72.1% (95% CI: 58.5–83.3%), adopting an RF + VI thickness cut-off of ≤29.2 mm, with 76.9% sensitivity and 63.3% specificity.

The statistical power of the accuracy analyses based on the AUC was above 80% in all analyses, except for the female subgroup in Figure 2, which showed a power of 75.6% (Table 2).

Table 3 summarizes the US cut-off points for sarcopenia diagnosis, using CT-based SMA as the gold standard. These cut-off points show AUC values indicating good accuracy (70–90%) and statistically significant (*p* < 0.05).

Although the US cut-off values for sarcopenia diagnosis differ between sexes and from the total sample, the differences are not significant, as their AUC percentages overlap within the 95% confidence intervals.

## 3. Discussion

The decline in physical functions and changes in muscle mass that occur with aging have long been studied. However, the term “sarcopenia” first appeared in the literature in 1989 to draw attention to the issue and gain recognition from the health authorities [34]. Since then, the understanding of the disease has evolved substantially [9,18,35]. This growing recognition led to the inclusion of a diagnostic code for sarcopenia (ICD-10 M62.84), formally acknowledging it as a muscle disease in 2016 [36]. However, some studies show that awareness of sarcopenia among healthcare professionals remains limited, and few institutions have established protocols for its diagnosis and management [35,37,38].

To mitigate the disease’s adverse clinical outcomes, early diagnosis with targeted interventions is essential [39]. In this context, opportunistic (timely) analysis can be effective in reducing disease burden [40]. This approach provides an early, rapid, and cost-effective means of diagnosis, making it a promising tool for assessing and managing sarcopenia in older adults [20,33,41,42].

To facilitate opportunistic sarcopenia analysis and early detection using US, we propose cut-off points for the thickness of the anterior thigh muscles. These cut-off points, obtained from RF and RF + VI US measurements, were validated against SMA assessed by CT as the reference standard, demonstrating good diagnostic accuracy [43], demonstrating good accuracy.

Studies by Chen et al. [44], Ozturk et al. [45], and Rustani et al. [46] also aimed to validate US for muscle assessment to diagnose sarcopenia in older adults using RF thickness; however, none used CT as the reference standard. Instead, they relied on DEXA, bioelectrical impedance analysis (BIA), and mid-arm muscle circumference, respectively.

A scoping review published by Staempfli et al. [33], guided by the question “*What is the current evidence on the validity of US for muscle assessment in diagnosing sarcopenia in older adults?*”, included six publications encompassing 24 validity tests. In these tests, CT was not used as the reference standard, and RF was the most frequently assessed muscle (in seven studies), with muscle thickness being the most studied parameter.

A systematic review with meta-analysis conducted by Fu et al. [32] aimed to summarize current knowledge on US accuracy for sarcopenia diagnosis. Seventeen studies were included, showing considerable heterogeneity in terms of countries, age ranges, population characteristics (none from South America), disease status, US frequencies (4 to 12 MHz), probe positioning (transverse or combined transverse-longitudinal), and the body side assessed.

In contrast, our study included Brazilian older adults of both sexes who underwent elective abdominal CT scans, independent of the underlying condition. US was performed on the right thigh using a 10 MHz linear transducer, positioned perpendicular to the limb’s axis between the anterior superior iliac spine and the medial femoral condyle.

Among studies using RF thickness measured by US, the highest AUC (92%, sensitivity 90.9%, specificity 92.2%) was reported by Sri-On et al. [47] in older Thai adults, using the BIA and EWGSOP2 diagnostic criteria [18] as comparators. The suggested cut-off points for sarcopenia diagnosis were ≤11 mm for men and ≤10 mm for women. In this study, RF thickness was measured on the right thigh between the anterior superior iliac spine and the superior lateral femoral epicondyle, with the transducer perpendicular to the thigh’s transverse axis.

Rustani et al. [46] conducted a study with elderly patients in an Italian hospital. They proposed RF thickness cut-off points of ≤9 mm for men and ≤7 mm for women to assess sarcopenia using US. These values demonstrated 100% sensitivity and 64% specificity, with AUCs of 94% for men and 92% for women. RF thickness was measured between the greater trochanter and the lateral epicondyle with the patient in the supine position, and the transducer oriented along the longitudinal axis.

The use of different measurement methods partially explains the discrepancies between the cut-off points reported in other studies and those proposed in our research. In our study, the male RF thickness cut-off of ≤19.1 mm had an AUC of 85.5%, with 81.2% sensitivity and 81.2% specificity. For females, the RF thickness cut-off of ≤15.9 mm yielded an AUC of 79.3%, with 84.6% sensitivity and 65.5% specificity. Furthermore, RF thickness was a better parameter than RF + VI combined.

Although other studies have assessed the US for sarcopenia diagnosis, comparing results has limitations. These include differences in the muscles measured and insufficient methodological details on US measurement techniques and parameters. Another factor to consider is the study population, as some studies include only patients with specific diseases.

Multiple methodological and biological factors may explain the differences in the proposed cutoff points. Differences in ultrasonographic measurement techniques—including the type of transducer, patient positioning, and anatomical site of measurement—can significantly influence the obtained values. In addition, the ethnic and anthropometric characteristics of different populations, considering height, body mass, and body composition, also contribute to variations in muscle thickness values. Finally, the use of varying reference methods for sarcopenia validation, such as CT, BIA, or DXA, and the application of distinct international diagnostic criteria, may further lead to variability in the proposed cutoff points across studies.

In a prospective study of 94 individuals with a mean age of 75.6 years, RF thickness was measured, and the risk of sarcopenia was 11.9 times higher for RF thickness < 15.4 mm. For this cut-off, sensitivity and specificity were 68.8% and 65.4%, respectively [48]. A similar value was found in our study, with a female RF thickness cut-off of ≤15.9 mm.

Rao et al. [49] conducted a study with 117 Indian patients with chronic kidney disease to propose thigh muscle thickness cut-offs for low muscle mass. The appendicular skeletal muscle index (ASMI) from BIA was used as the reference. They reported 11 mm for men (sensitivity 76%, specificity 64%, AUC = 0.749) and 10 mm for women (sensitivity 70%, specificity 55%, AUC = 0.628). In our study, the cut-off values were higher; however, their sample included patients with chronic kidney disease, no age restriction, and a diverse ethnic background, which partly explains the differences.

Fukumoto et al. [50] conducted a cross-sectional study with 204 older adults (mean age, 75.4 years) and 59 young adults (mean age, 22.3 years). They found that the RF thickness cut-offs, based on two standard deviations below the young adult mean, were 18.5 mm for men and 14.2 mm for women, with low muscle mass prevalence of 69.4% in men and 36.7% in women. RF thickness was defined as the distance between the superficial and deep muscle fascia, measured from the lateral epicondyle to the greater trochanter, with participants seated and hips flexed at 90°. Sri-On et al. [47] note that RF isometric contraction depends on knee and hip angles during extension; thus, patient positioning affects RF thickness measurements. This suggests that studies and cut-off points should only be compared if the patient’s position is consistent.

Eşme et al. [51] analyzed 40 patients (mean age 53.2 years) and found a positive correlation between handgrip strength and RF cross-sectional area. Despite the differences between our study and theirs, they reported a 76% sensitivity and 69% specificity for RF cross-sectional area in predicting sarcopenia, supporting its diagnostic utility.

de Luis Roman et al. [52] conducted a prospective multicenter cohort study in Spain to evaluate RF US for detecting sarcopenia in hospitalized patients at malnutrition risk. RF cross-sectional area correlated with both body mass (BIA) and handgrip strength. They showed that RF US can predict sarcopenia and aid nutritional assessment in clinical practice. Although our analysis focused on RF thickness rather than area, results can be partially extrapolated, as muscle area correlates with thickness.

Table 4 presents the ultrasonographic cutoff points proposed in this study for diagnosing sarcopenia in Brazilian older adults, compared with values suggested in previous studies conducted in different populations. This comparison highlights methodological and population differences that influence the reference values for muscle thickness.

Due to the lack of Brazilian population-based cut-off points for sarcopenia diagnosis, we opted to use lumbar (L3) CT to measure muscle mass as a reference to validate the anterior thigh muscle thickness obtained by US. As previously mentioned, some studies did not use this gold standard method (CT) to validate RF thickness cut-off points. The use of other methods is valid; however, it partially hinders comparisons with previous research.

The results of this study propose ultrasonographic cutoff points specific to the Brazilian older adult population, which demonstrated acceptable diagnostic accuracy for sarcopenia when compared with values established in other populations. These findings reinforce the importance of standardizing national parameters, taking into account ethnic and methodological differences that influence muscle thickness. In clinical practice, the suggested cutoff points can aid in the early identification of sarcopenia and inform the development of targeted nutritional and functional interventions. Furthermore, this study provides a foundation for future multicenter research that may validate and expand these findings in more representative samples of the Brazilian population.

Another critical factor is that US image acquisition depends on the operator and device settings [31]. Therefore, we suggest that future studies testing the accuracy of US cut-off points for sarcopenia diagnosis should be conducted by the same operator and, if possible, using devices with similar settings to reduce methodological bias in cross-study comparisons. Although all measurements in this study were performed by a single radiologist, ensuring procedural consistency, future studies should include interobserver reliability analyses to strengthen the reproducibility of the findings. Assessing variability among examiners is essential to confirm the applicability of the cutoff points across different clinical settings and levels of professional experience, thereby promoting the standardization of ultrasonographic measurements and their broader adoption in clinical practice.

In a systematic review and meta-analysis by Fu et al. [32], of the 17 included articles, ten assessed RF, with some using RF thickness and others using RF cross-sectional area. In this review, ten studies used low muscle mass as the sole criterion for sarcopenia, partly due to the lack of a universal consensus at the time. We believe this does not preclude comparisons between studies. However, caution is advised, as diagnostic bias may occur: the same individual could be classified as sarcopenic under one criterion but not another, potentially affecting the cut-off value obtained.

Petermann-Rocha et al. [53] conducted a study on the global prevalence of sarcopenia using data from 263 systematic reviews and 163 meta-analyses. They concluded that different classification criteria and cut-off points resulted in sarcopenia prevalence ranging from 0.2% to 86.5% in systematic reviews and 10% to 27% in meta-analyses, highlighting the need for standardization of diagnostic criteria.

Stuck et al. [54] conducted a European study in community-dwelling older adults across several countries. Using 12 sarcopenia definition criteria, they found discrepancies in prevalence at both population and individual levels, demonstrating the need for uniform diagnostic criteria to validly and reliably identify individuals with compromised muscle health.

Although international organizations such as EWGSOP2 and the Asian Working Group for Sarcopenia (AWGS) do not yet recommend the US for sarcopenia assessment and diagnosis, recent studies highlight its potential utility [22]. This underscores the need for multicenter studies to establish population-specific cut-off points, thereby improving accuracy in identifying patients at risk or with established disease. US is widely available, safe, cost-effective, and shows good inter- and intra-observer reproducibility. It can be used in a broad range of settings, including at the bedside, especially when compared with imaging methods considered gold standards for sarcopenia diagnosis, such as CT, MRI, and DEXA [22,23,24,25].

In our study, we chose to evaluate anterior thigh muscle thickness (RF and RF + VI) because these are postural, load-bearing muscles, easily accessible and affected early by sarcopenia [55].

Our study was limited to a quantitative assessment of anterior thigh muscle thickness (B-mode) only, which represents one of its potential limitations. Existing literature on US muscle assessment has focused primarily on parameters related to muscle quantity, such as muscle thickness, fascicle length, and pennation angle [56,57,58]. However, muscle quality—not just quantity—is increasingly recognized as an independent variable affecting clinical outcomes [59].

Muscle tissue composition changes with aging, characterized by fat infiltration, altered biomechanical properties, and a consequent reduction in muscle function. These qualitative changes can be assessed by shear-wave elastography, a promising tool for measuring muscle tissue composition (“stiffness”) [60]. Elastography is currently available on a limited number of devices due to cost and the need for specialized operator training, which represents a limitation. Nevertheless, this landscape is expected to change, providing valuable information as its use becomes more widespread.

Finally, new technologies employing artificial intelligence have emerged, showing promising results for sarcopenia assessment [61].

## 4. Materials and Methods

### 4.1. Study Design

This is an observational, analytical, cross-sectional accuracy study conducted at an imaging diagnostic center in Marília, São Paulo, Brazil.

### 4.2. Ethical Aspects

The research project was approved by the Research Ethics Committee on Human Subjects of the University of Marília–UNIMAR (approval number 6,269,555) and followed the World Medical Association’s Declaration of Helsinki—Ethical Principles for Medical Research Involving Human Subjects [62].

### 4.3. Sample

The sample size was estimated using MedCalc software (version 22.0) to validate the use of US for diagnosing sarcopenia through Area Under the Curve (AUC) analysis. Considering a type I error margin of 5%, a study power of 80%, a null hypothesis AUC of 50%, an alternative hypothesis AUC of 80%, and a negative/favorable ratio of 1, the minimum sample size was 26 participants, allowing for sex-specific analysis. For this study, 88 patients aged 60 years or older, of both sexes, undergoing elective abdominal CT scans at the center as mentioned earlier, and eligible for inclusion, were invited to participate. Those who consent provided written informed consent. Exclusion criteria included patients scheduled for emergency surgery on the same day as the CT, victims of abdominal trauma or fractures, prior abdominal or lumbar spine surgeries, extensive burns, conditions preventing ambulation, lower limb amputations, as well as patients receiving palliative care or under isolation due to infectious causes. Included patients, in addition to the planned CT, also underwent musculoskeletal US evaluation of the thigh, performed on the same day by the same radiologist.

### 4.4. Computed Tomography

The CT study was performed using a 64-slice GE scanner, acquiring images of the abdomen at the level of the third lumbar vertebra (L3) in the non-contrast phase to measure SMA [63,64]. The cross-sectional area of all muscles at this level was used, with a threshold between −30 and 150 Hounsfield units (HU) [65]. CT measurements were performed manually by the same operator, a biomedical professional, and verified by the same radiologist.

### 4.5. Ultrasound

US images were acquired using a Toshiba Aplio A system with a 5–10 MHz linear transducer in B-mode. Images were obtained from the anterior compartment of the right thigh, with the patient in a supine position and hips and shoulders in neutral alignment. The transducer was positioned perpendicular to the long axis of the limb, at two-thirds of the distance between the anterior superior iliac spine and the medial femoral condyle.

For imaging the rectus femoris (RF), the reference line was established at the most central point, excluding its fascia, with the tracing extending from the highest point of the femur to the end of the RF fascia. For the vastus intermedius (VI), a straight line was used to quantify its thickness, starting at the highest point of the femur and ending at the end of the muscle, excluding the muscular fascia. This technique followed the recommendations of Perkisas et al. [66,67].

From the US images, the thickness of the RF muscle and of the combined RF + VI was measured [30].

### 4.6. Analysis Methodology

Based on CT, sarcopenia was defined according to the criteria proposed by Derstine et al. [43], which establishes an SMA of less than 144.3 cm^2^ for men and 92.2 cm^2^ for women. Data obtained from the US and CT were cross-analyzed to propose reliable US cut-off points for diagnosing sarcopenia in the study sample, using RF and RF + VI thickness measurements. Quantitative variables were described as mean ± standard deviation (SD). Normality was assessed using the Kolmogorov–Smirnov test with Lilliefors’ correction. Comparisons of means were performed using Student’s *t*-test or the Mann–Whitney test, as appropriate. Receiver Operating Characteristic (ROC) curves were used to determine the sensitivity and specificity of independent variables (US measurements) for sarcopenia diagnosis, as well as positive and negative predictive values. The area under the curve (AUC) and 95% confidence intervals (CI) were calculated. The optimal cut-off point was determined using the confidence interval of Youden’s index. A significance level of 5% (*p* ≤ 0.05) was adopted, and analyses were conducted using SPSS version 24.0 for Windows. ROC curve analyses were performed using MedCalc version 22.0. The accuracy of the US in diagnosing sarcopenia was evaluated according to AUC, following these criteria: AUC > 90% indicated excellent accuracy; AUC between 90% and 70% showed good accuracy; AUC between 70% and 50% indicated acceptable accuracy; and AUC < 50% indicated unacceptable accuracy [68].

To estimate the statistical power of the accuracy analyses based on the AUC, a one-sided test for comparing proportions was used, employing the NormalIndPower function from the statsmodels package (Python 0.14.4), which is based on the standard normal distribution. The effect size was calculated as the difference between the observed AUC and the null AUC (0.5), standardized by the theoretical range of the AUC (0.5). The following parameters were considered: a significance level of 5% (α = 0.05), a one-sided alternative hypothesis (AUC > 0.5), a 1:1 group ratio, and the specific sample sizes for each subgroup (total, male, and female).

## 5. Conclusions

Examining skeletal muscles using US examinations is a safe and accessible method with potential to become valuable in diagnosing sarcopenia, to improve risk stratification, and facilitate the clinical risk decision-making process regarding nutritional interventions against this critical condition. We identified cut-off points with good AUC values to suggest sarcopenia diagnosis by US or for screening purposes in both Brazilian men (≤19.1 mm for RF thickness and ≤31.9 mm for RF + VI thickness) and women (≤15.9 mm for RF thickness and ≤29.2 mm for RF + VI thickness), particularly when focusing solely on RF thickness assessment.

For clinical application, we propose a two-step screening approach: (1) use RF thickness measurement as an initial screening tool in primary care or geriatric clinics; and (2) refer patients who fall below the proposed cut-offs for a comprehensive sarcopenia assessment following EWGSOP2 criteria, including muscle strength and physical performance tests. This approach could facilitate early detection of at-risk individuals while maintaining diagnostic accuracy through subsequent comprehensive evaluation

Multicenter studies with larger sample sizes are needed to confirm the findings reported in this study.

## Figures and Tables

**Table 1:** Thickness of the rectus femoris (RF) and rectus femoris plus vastus intermedius (RF + VI) muscles measured by ultrasound according to sarcopenia diagnosis (n = 88).

	RF Thickness (mm)	RF + VI Thickness (mm)
	Mean ± Standard Deviation (SD)	
Diagnosis based on skeletal muscle area (cm^2^)		
With sarcopenia (n = 42)	14.6 ± 2.8	27.2 ± 5.0
Without sarcopenia (n = 46)	18.3 ± 4.2	33.2 ± 7.1
*p*-value	<0.0001 *	<0.0001 *

* Mann–Whitney test for independent samples. RF = rectus femoris; RF + VI = rectus femoris plus vastus intermedius.

**Table 2:** Statistical power of the accuracy analyses based on the area under the curve (AUC) for each subgroup (total, male, and female).

Subgroup	Observed AUC	Sample Size (n)	Effect Size	Statistical Power
Figure 1-Total	0.780	88	0.560	0.981
Figure 1-Male	0.855	31	0.710	0.875
Figure 1-Female	0.793	56	0.586	0.927
Figure 2-Total	0.745	88	0.490	0.946
Figure 2-Male	0.859	31	0.718	0.881
Figure 2-Female	0.721	56	0.442	0.756

**Table 3:** Proposed cut-off points for sarcopenia diagnosis using ultrasound compared with computed tomography, based on skeletal muscle area.

	Total Participants (n = 88)	Male Participants (n = 32)	Female Participants (n = 56)	*p*-Value
Reference: skeletal muscle area (cm^2^)				
RF thickness (mm)	≤16.6	≤19.1	≤15.9	0.4934
RF + VI thickness (mm)	≤31.9	≤31.9	≤29.2	0.1573

RF = rectus femoris; RF + VI = rectus femoris plus vastus intermedius.

**Table 4:** Table of sarcopenia cut-off points proposed in studies and their methodological characteristics.

Reference	Measurement	Reference Standard	Reference Population	Cut-Off Points for Sarcopenia	AUC (Sensitivity and Specificity)	Positioning During the Exam
Our study	RF thickness	CT	Brazilian	Men: ≤19.1 mm	85.5% (81.2% and 81.2%)	Right thigh, patient in supine position, with a 10 MHz linear transducer placed perpendicular to the longitudinal axis of the limb, between the anterior superior iliac spine and the medial condyle of the knee.
				Women: ≤15.9 mm	79.3% (84.6% and 65.5%)	
Rustani et al. [46]	RF thickness	CAMA	Italian	Men: ≤9 mm	94% (100% and 64%)	Between the lateral epicondyle and the greater trochanter, participant in the supine position, with the transducer along the longitudinal axis.
				Women: ≤7 mm	92% (100% and 64%)	
Barotsis et al. [48].	RF thickness	DEXA	Greek	15.4 mm	67% (68.8% and 65.4%)	Halfway along the line from the anterior–superior iliac spine to the superior pole of the patella. The transducer was placed perpendicular to the skin and slightly angled (elevationally) to achieve the brightest echo from the muscle fascia. Cross-section taken on the non-dominant side.
Fukumoto et al. [50]	RF thickness	BIA	Japanese	Men: 15.1 mm	77.5% (69.2% and 83.7%)	Participant seated with 90° flexion at the hips and knees. The transducer is positioned at the midpoint between the greater trochanter and the lateral condyle of the femur, placed perpendicular to the longitudinal axis.
				Women: 14.3 mm	65.4% (60% and 67%)	
Ozturk et al. [45]	RF thickness	BIA	Not reported	Men: ≤15.5 mm	76% (80% and 61%)	Assessed at the midpoint between the anterior superior iliac spine and the proximal border of the patella.
				Women: ≤13 mm	73% (88.2% and 60.6%)	
Sri-On et al. [47]	RF thickness	BIA and EWGSOP2 diagnostic criteria	Thai	Men: ≤11 mm	92% (90.9% and 92.2%)	Right thigh, between the anterior superior iliac spine and the upper lateral epicondyle of the femur, with the transducer placed perpendicular to the transverse axis of the thigh.
				Women: ≤10 mm		
Rao et al. [49]	Thigh muscle thickness	BIA	Indian	Men: 11 mm	74.9% (76% and 64%)	Participant seated with hip and knee joints at 90°. Measurement taken at the midpoint between the greater trochanter and the patella, using a curvilinear transducer positioned perpendicular to the anterior surface of the thigh. RF thickness is measured between the anterior and posterior fascia of the rectus femoris.
				Women: 10 mm	62.8% (70% and 55%)	
				Women: 14.3 mm	65.4% (60% and 67%)	
Esme et al. [51]	Cross-sectional area	BIA	Not reported	5.65 mm^2^	72.8% (76% and 69%)	Right side, at the midpoint between the anterior superior iliac spine and the upper edge of the patella.
Luis Roman et al. [52]	Cross-sectional area of the rectus femoris	BIA	Spanish	Men: 9.66 cm^2^	77.5% (78.6% and 69.7%)	On the right side, using a portable ultrasound. Participant in supine position, knees extended and relaxed. A 7.5–10 MHz linear ultrasound probe was used. The measurement site was two-thirds of the femur length, between the anterior superior iliac spine and the upper border of the patella. The transducer was positioned perpendicular to the longitudinal axis of the thigh.
				Women: 10.4 cm^2^	53.4% (74.5% and 35.4%)	

RF = rectus femoris; CT = computed tomography; BIA = bioelectrical impedance analysis; EWGSOP2 = European Working Group on Sarcopenia in Older People 2; DEXA = dual-energy X-ray absorptiometry; CAMA = corrected arm muscle area (used as reference for muscle mass).

## Data Availability

The raw data supporting the conclusions of this article will be made available by the authors on request.
